# Incorporation of Rutin in Electrospun Pullulan/PVA Nanofibers for Novel UV-Resistant Properties

**DOI:** 10.3390/ma9070504

**Published:** 2016-06-23

**Authors:** Yongfang Qian, Mengjie Qi, Laijiu Zheng, Martin W. King, Lihua Lv, Fang Ye

**Affiliations:** 1 School of Textile and Material Engineering, Dalian Polytechnic University, Dalian 116034, China; qianyf@dlpu.edu.cn (Y.Q.); qimengjie123@gmail.com (M.Q.); fztrxw@dlpu.edu.cn (L.Z.); lvlh@dlpu.edu.cn (L.L.); 2 College of Textiles, North Carolina State University, Raleigh, NC 27695, USA; martin_king@ncsu.edu

**Keywords:** pullulan, rutin, electrospinning, nanofiber, UV resistance

## Abstract

This study aimed to investigate the incorporation of rutin into electrospun pullulan and poly(vinyl alcohol) (PVA) nanofibers to obtain ultraviolet (UV)-resistant properties. The effect of weight ratios between pullulan and PVA, and the addition of rutin on the nanofibers’ morphology and diameters were studied and characterized by scanning electron microscopy (SEM). Fourier transform infrared (FTIR) analysis was utilized to investigate the interaction between pullulan and PVA, as well as with rutin. The results showed that the inclusion of PVA results in the increase in the fiber’s diameter. The addition of rutin had no obvious effect on the fibers’ average diameters when the content of rutin was less than 7.41%. FTIR results indicated that a hydrogen bond formed between pullulan and PVA, also between these polymers and rutin. Moreover, the addition of rutin could enhance the mechanical properties due to its stiff structure and could decrease the transmittance of UVA and UVB to be fewer than 5%; meanwhile, the value of ultraviolet protection factor (UPF) reached more than 40 and 50 when the content of rutin was 4.46% and 5.67%, respectively. Therefore, the electrospun pullulan/PVA/rutin nanofibrous mats showed excellent UV resistance and have potential applications in anti-ultraviolet packaging and dressing materials.

## 1. Introduction

Electrospinning is a simple and effective method to fabricate nonwoven mats with large surface areas and porosities [1,2]. In this method, high voltage is applied on the drop to the tip of the needle as the draft force on the polymer solution or melt. When the applied electrical force overcomes the critical surface tension of the polymer liquid, the liquid is ejected from the nozzle, stretched, and finally deposited on the grounded collector in the form of nonwoven mats with fibers ranging from tens of microns to nanometers in diameter [3]. The effected parameters on the fiber formation and diameters in the electrospinning process involve characteristics of the solution (e.g., electric conductivity, viscosity, surface tension, and concentration), controlled variables (e.g., tip-to-collector distance, voltages, and feeding rate), and the atmosphere (mainly the humidity and the temperature) [4]. 

Pullulan is an extracellular microbial polysaccharide produced by the yeast-like fungus *Aureobasidium pullulans* [5,6]. The basic chemical structure is a linear maltotriose unit connected by α-1,6 linkages, while the internal glucose unit within maltotriose is a α-1,4 glycosidic linkage. Remarkably, this structure gives pullulan excellent solubility and high resilience in terms of structure [7]. Islam et al. [8] and Karim et al. [9] investigated the incorporation of PVA and montmorillonite into pullulan nanofibers to improve the mechanical properties and thermal performance of electrospun nanofibrous mats. Islam et al. [10] and Karim et al. [11] studied functionalized pullulan with fluorinated silane to fabricate super hydrophobic membranes. 

Poly(vinyl alcohol) (PVA) is a highly biocompatible and nontoxic polymer with good water-solubility, which is influenced by the alcoholysis degree and degree of polymerization. The unique properties lead to the use of PVA in a wide range of applications such as the food, packaging, cosmetic, spinning, and paper-making industries [12]. PVA is usually used as the strong part to improve or modify the physicochemical properties in electrospinning. Mohmoodi et al. [13] found that the addition of PVA to the solution of chitosan can improve the spinnability; moreover, the resultant nanofibrous mats have good absorbent ability and can be applied in the removal of dye from colored wastewater. Sousa et al. [14] introduced PVA to the agar solution, and Wang et al. [15,16] incorporated PVA into honey and milk to improve the spinnability. In addition, Wang et al. [17] demonstrated that incorporation of pleurocidin into electrospun PVA nanofibers can preserve the bioactivity of pleurocidin and realize sustained release to improve food safety. 

Rutin, 3′,4′,5,7-tetrahydroxyflavone-3β-d-rutinoside, is one of the most abundant natural flavonoids and has many biological properties, being anti-inflammatory, antiallergenic, and antimicrobial [18,19]. Rutin was found to be photo-stable and capable of enhancing the defense system against environment stresses, including low temperature, UV light, and desiccation [20,21]. Xing et al. [22] proposed that rutin be added to poly(l-lactide-*co*-glycolide) (PLGA) solution and then electrospun to obtain antibacterial nanofibrous mats. However, reports related to the UV resistance of rutin-contained nanofibrous mats are scarce. This study aimed to investigate the incorporation of rutin into the pullulan- and PVA-blended nanofibers. The morphologies of electrospun pullulan and PVA nanofibers, and the effect of introducing rutin onto the formed fibers, were observed via scanning electron microscopy (SEM). Fourier transform infrared (FTIR) spectrophotometer was utilized to analyze the interaction between pullulan and PVA, as well as the interaction between these polymers and rutin. Finally, the ultraviolet (UV)-resistant properties were tested with a UV performance tester. 

## 2. Results and Discussion

### 2.1. Morphology of Electrospun Pullulan and PVA Nanofibers

**Figure 1:**
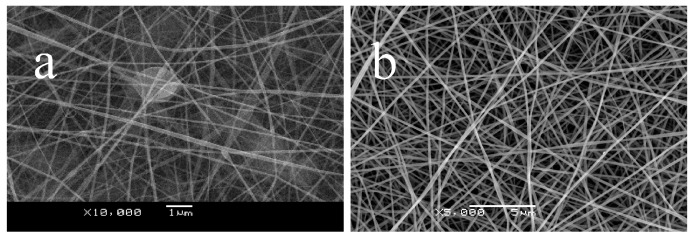

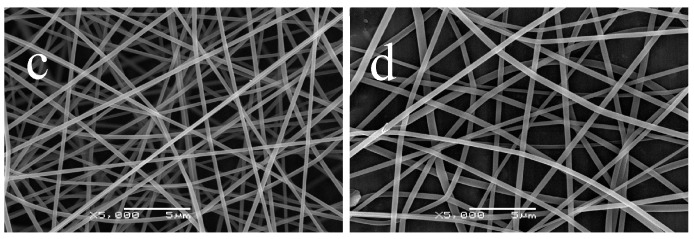
Scanning electron microscopy (SEM) micrographs of electrospun pullulan/poly(vinyl alcohol) (PVA) blended fibrous mats at a concentration of 0.15 g/mL with weight ratios of (**a**) 100/0; (**b**) 75/25; (**c**) 50/50; (**d**) 25/75.

The concentration or the corresponding viscosity was one of the most effective variables for controlling the fiber morphology [23]. A higher concentration results in higher viscosity. Fibers with different weight ratios of pullulan and PVA (Figure 1) at the same total concentration of 0.15 g/mL show smooth and uniform morphology. When the contents of the PVA increased from 0%, 25%, 50%, to 75%, the average diameters were 89, 171, 311 and 413 nm, respectively. The diameters of the fibers increased due to the increased viscosity. Pure PVA failed to be electrospun at a concentration of 0.15 g/mL since the solution was too viscous to be drafted. Therefore, the incorporation of PVA was capable of improving the spinnability of pullulan.

### 2.2. The Effect of Rutin on Electrospun Pullulan and PVA Nanofibers

**Figure 2:**
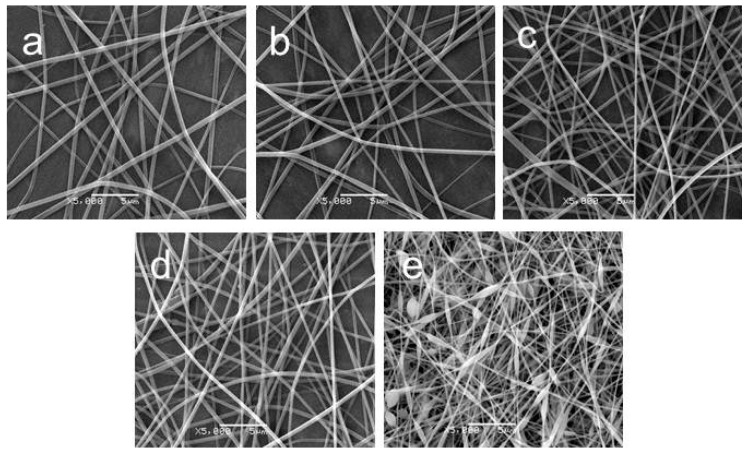
SEM micrographs of electrospun pullulan/PVA nanofibers containing different amounts of rutin (*w*/*w*): (**a**) 3.23%; (**b**) 4.46%; (**c**) 5.67%; (**d**) 7.41%; (**e**) 8.54%.

The average diameter of an electrospun pullulan and PVA nanofibrous mat with a weight ratio of 50/50 was 311 nm. When rutin was added to the spinning solution and then electrospun, the collected nanofibrous mats became light yellow compared with the original white mats. The morphologies of electrospun pullulan and PVA nanofibers containing different amounts of rutin are shown in Figure 2. When the contents of rutin were 3.23%, 4.46%, 5.67%, and 7.41% (*w*/*w*) compared to pullulan and PVA blends, the average diameters were 312, 322, 326 and 334 nm, respectively, which indicated that the incorporation and the amount of rutin had no obvious influence on the morphology or the diameters of the electrospun nanofibers. However, when the content of rutin reached 8.54% (*w*/*w*), the morphology exhibited spindle-like beads between the fibers, which means that the spinnability had been affected. It is noted that the rutin cannot be fabricated into any type of fiber without other polymer materials. Therefore, there would be no fibers collected on the grounded plate if the weight ratio of rutin exceeded a limited value.

### 2.3. FTIR Analysis of Electrospun Pullulan/PVA and Pullulan/PVA/Rutin Nanofibers

**Figure 3:**
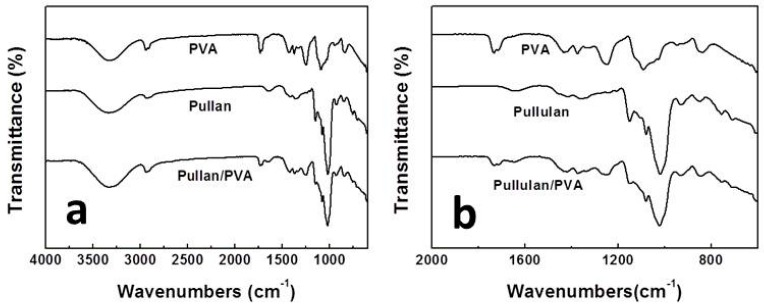
Fourier transform infrared (FTIR) spectra of PVA, pullulan and blended nanofibrous mats in wavenumbers range of (**a**) 4000–600 cm^−1^ and (**b**) 2000–600 cm^−1^.

The FTIR spectra gave information about information about the structure and interaction of the blended membranes studied. The spectra of pure pullulan, PVA, and pullulan-/PVA-blended membranes in the range of 4000–600 cm^−1^ (Figure 3a) and 2000–600 cm^−1^ (Figure 3b) are shown. Pure PVA membrane exhibits an identical strong absorption peak located at 1089 cm^−1^, indicating the presence of C–O. The absorption peak at 2939 cm^−1^ is due to the stretching vibration of the CH_2_ group. The broad absorption peak at 3330 cm^−1^ is attributed to the stretching vibration of hydroxyl group (–OH). The spectrum of pure pullulan has a strong absorption band at 848 cm^−1^, which is characteristic of the α-glucopiranosid units. The band at 754 cm^−1^ proves the presence of α-(1,4) glucosidic bonds, and the band at 930 cm^−1^ demonstrates the presence of α-(1,6) glucosidic bands [8,9]. The band at 2930 cm^−1^ is caused by the stretching vibrations of methyl or methylene groups. Bands attributed to CH/CH_2_ deformation vibrations occur in the range of 1300–1500 cm^−1^. The broad adsorption peak at 3330 cm^−1^ is also assigned to the hydroxyl group (–OH). Comparing the spectra of pure pullulan and PVA, the absorption peak of the hydroxyl group (–OH) in the spectra of the electrospun pullulan/PVA blend shifts from 3330 to 3320 cm^−1^, which is evidence that a hydrogen bond formed between the pullulan and PVA molecules. Therefore, the FTIR spectroscopy is an effective way of examining the interaction between polymers. 

**Figure 4:**
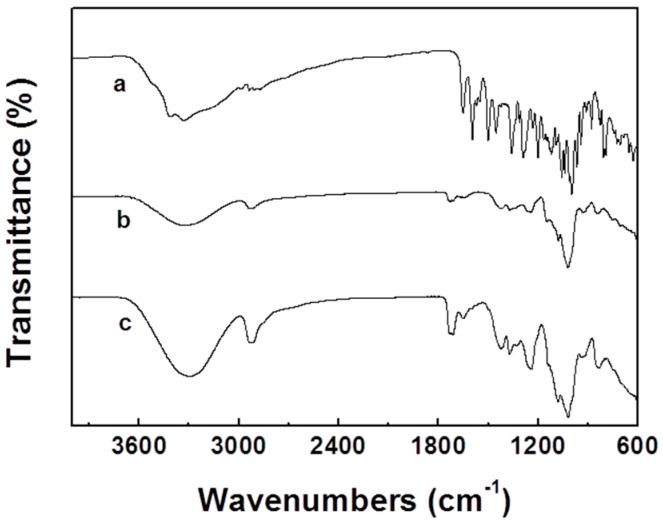
FTIR spectra of the rutin (**a**), electrospun pullulan/PVA (**b**) and pullulan/PVA/rutin (**c**) nanofibrous membranes.

Figure 4 shows the spectra of rutin, the electrospun pullulan/PVA, and the pullulan/PVA/rutin membranes. The spectrum of the rutin has absorption peaks at 3330 and 1359 cm^−1^, corresponding to the O–H stretching vibrations of intercalated water. The adsorption peak located at 1650 cm^−1^ is due to the carbonyl stretching vibration. The peaks located at 1203, 1123 and 1089 cm^−1^ represent a C–O–C bond stretching in the ethyl dioxy ring deformation. The peaks at 1504 and 1359 cm^−1^ are attributed to the asymmetric stretching of C=C bond and the inter-ring stretching of C–C. The peak located at 1057 cm^−1^ is due to the epoxide group stretching. When rutin was added into the pullulan/PVA nanofibers, the FTIR spectra of the electrospun pullulan/PVA/rutin membrane exhibited a slight change compared with that of pullulan/PVA. The absorption band of the hydroxyl group became stronger and shifted from 3320 to 3306 cm^−1^, which also indicated the formation of hydrogen between rutin and the pullulan/PVA molecules. 

### 2.4. Mechanical Properties of PVA/Pullulan and PVA/Pullulan/Rutin Nanofibrous Mats

The stress and strain values of electrospun pullulan and PVA nanofibrous mats at different weight ratios are summarized in Table 1. When the weight percentage of PVA was 0%, 25%, 50%, and 75%, the average ultimate tensile stress was 1.07, 1.72, 1.94, and 2.56 MPa, while the average strain was 31%, 28%, 25%, and 19%, respectively. The average stress increased as the content of PVA increased, but the average strain decreased. Synthetic polymers usually have better spinnability and mechanical properties; thus, the incorporation of synthetic polymers with natural materials is an effective way of enhancing the formation of fibers and mechanical properties [24,25].

The effect of incorporating rutin into the mechanical properties of electrospun pullulan/PVA nanofibrous mats are summarized in Table 2. When the weight percentage of rutin was 0%, 3.23%, 4.46%, 5.67%, and 7.41%, the average ultimate tensile stresses were 1.94, 2.38, 2.85, 3.22, and 3.1 MPa, and the average stains were 25%, 23%, 21%, 16%, and 12%, respectively. It is noted that the electrospun pullulan/PVA/rutin nanofibrous mats had greater tensile stress than that of pullulan/PVA, and the ultimate tensile strength exhibited an increasing trend as the content of rutin increased, which indicated that the addition of rutin was capable of enhancing the mechanical properties of the nanofibrous mats. Rutin has stiff aromatic rings and ethylene dioxy rings in its structure [26]; meanwhile, rutin was capable of randomly dispersing in the electrospun nanofibers [22]. Thus, the addition of a limited amount of rutin can enhance the strength to some extent. Combined with the SEM morphology, the incorporation of rutin into the nanofibers can enhance the mechanical properties but has no obvious influence on the formation and diameter of fibers. 

### 2.5. The Ultraviolet-Resistant Property of the Electrospun Nanofibrous Mats Containing Rutin

UV radiation consists of ultraviolet A (UVA), ultraviolet B (UVB), and ultraviolet C (UVC), all of which represent the regions 315–400 nm, 280–315 nm, and 200–280 nm, respectively. Excessive exposure to solar UVA and UVB radiation can cause skin cancers [20]. UVA and UVB transmittances, as well as ultraviolet protection factor (UPF), were tested with a spectrophotometer, and the results are summarized in Table 3. For pure rutin, the UVA and UVB transmittances, as well as the UPF, were 0.34 ± 0.17, 0, and more than 50, respectively. Generally, the transmittances of UVA and UVB decreased while the UPF markedly increased and the content of rutin increased. When the addition of rutin was greater than 3.23%, the transmittances of UVA and UVB of electrospun pullulan/PVA/rutin nanofibrous mats were lower than 5%. The UPF was above 40 when the content of rutin was 4.46% and was more than 50 when the content was 5.67%. The UV-blocking effect was superior to traditional fabrics treated with ZnO or TiO_2_, of which the transmittance was less than 20% and the UPF was above 50 [27]. Both the UVA and UPF met the requirements and standards of UV-resistant products. The UV resistance of the electrospun nanofibrous mats is applicable to anti-ultraviolet packaging and dressing materials.

## 3. Materials and Methods 

### 3.1. Materials 

Pullulan, food grade, was purchased from Hayashibara Biochemical Laboratories Inc. (Okayama, Japan). PVA in analytical pure grade was obtained from Kelong Chemical Reagent Factory (Chengdu, China). Rutin was purchased from Aladdin Industrial Corporation (Shanghai, China) with a purity of 98%. Distilled water was used as the solvent to prepare all solutions. 

### 3.2. Preparation of the Spinning Solution and Electrospinning

Pullulan and PVA powder were dissolved together in distilled water with different weight ratios of 100/0, 75/25, 50/50, 25/75, and 0/100, separately, at total concentrations of 0.15 g/mL. Rutin was dissolved in a pullulan-/PVA-blended solution (50/50) with a weight percentage at 3.23%, 4.46%, 5.67%, 7.41%, and 8.54%, respectively, compared to the pullulan and PVA blends. All the solutions were stirred for 24 h at a temperature of 30 °C.

The blended polymer solutions were electrospun using a high voltage of 24 kV supplied by a high voltage power supply (JDF-1, Beijing, China) purchased from the BMEI Co., Ltd. The feeding rate of the solution was set at 0.6 mL/h and controlled by an accurate syringe driver (789100C, Cole-Parmer, Vernon Hills, IL, USA). The membranes of the nanofibers were collected with aluminum platinum paper, which was faced vertically to the needle tip with a tip-to-collector distance of 12 cm. The electrospinning process was conducted at a room temperature of 30 °C and a relative humidity of 45%.

### 3.3. Characterization of the Membranes 

The morphologies of electrospun nanofibers were observed via scanning electron microscopy (SEM, JSM 6040, JEOL, Tokyo, Japan) after being sprayed with platinum. The diameters were conducted by image visualization software Image J (National Institutes of Health, Bethesda, MD, USA). A Fourier transform infrared (FTIR) spectrophotometer (Nicolet Is5, Thermo Fisher Scientific, Waltham, MA, USA) was utilized to analyze the chemical structure and interaction between pullulan and PVA, as well as between these polymers and rutin, through the reflection mode with a nanofibrous membrane. All spectra were recorded at 1 cm^−1^ intervals in the scanning range of 4000–600 cm^−1^. The mechanical properties of the tensile stress and strain were tested by a tensile machine (Model YG061, Laizhou Electronic Equipment Company, Laizhou, China) at an ambient temperature of 22 °C and a relative humidity of 65%. The dimensions of the tested samples were 50 mm in length and 10 mm in width, while the gauge length between the two holders was 30 mm. The specimen thicknesses were measured using a micrometer having a precision of 0.01 mm. UV transmittance and UV protection factor (UPF) of the electrospun rutin contained nanofibrous mats that were tested with a Textile-UV-performance tester (YG(B)912E, Darong Textile Instrument, Wenzhou, China). The scanned wavenumber ranged from 280 to 400 nm (±0.5 nm). The UVA, UVB transmittance, and UPF were recorded automatically after scanning. 

## 4. Conclusions

Pullulan/PVA nanofibrous mats with and without rutin were fabricated via the electrospinning technique. The effect of total concentration, the weight ratio of pullulan/PVA, as well as the different addition amounts of rutin on the formation and diameters of nanofibers were studied. SEM results showed that the average diameter increased as the content of PVA increased. Moreover, the addition and the amount of rutin had no obvious influence on the average diameters when the content was less than 7.41% (*w*/*w*), but formed beaded fibers when the amount was more than 8.54% (*w*/*w*). FTIR analysis confirmed the hydrogen formation between pullulan and PVA molecules, as well as rutin and the pullulan/PVA blend. The mechanical properties showed that the tensile stress increased as strain decreased and the weight ratio of PVA increased. The addition of rutin could enhance the mechanical properties to some extent due to its stiff structure. From a UV-resistant properties test, the incorporation of rutin was able to decrease the transmittance of UVA and UVB to be less than 5%; meanwhile, the value of UPF was above 40 and above 50 when the contents of rutin were 4.46% and 5.67%, respectively. Therefore, the electrospun pullulan/PVA/rutin nanofibrous mats showed excellent UV resistance and can be used as anti-ultraviolet packaging and outdoor materials.

## Figures and Tables

**Table 1:** The tensile properties of electrospun pullulan and PVA nanofibrous mats with different weight ratios (*n* = 3).

Pullulan/PVA	100/0	75/25	50/50	25/75
Stress (MPa)	1.07 ± 0.062	1.72 ± 0.125	1.94 ± 0.09	2.56 ± 0.147
Strain (%)	31 ± 3.606	28 ± 4.583	25 ± 5.568	19 ± 2.646

**Table 2:** The tensile properties of electrospun nanofibrous mats with different weight percentages of rutin compared to pullulan/PVA (*n* = 3).

Rutin (%)	0	3.23	4.46	5.67	7.41
Stress (MPa)	1.94 ± 0.09	2.38 ± 0.274	2.85 ± 0.298	3.22 ± 0.368	3.1 ± 0.171
Strain (%)	25 ± 5.568	23 ± 2	21 ± 2.646	16 ± 2.646	12 ± 3

**Table 3:** The UVA, UVB transmittance and UPF of electrospun pullulan/PVA nanofibrous mats with different contents of rutin (*n* = 3).

Rutin (%)	0	3.23	4.46	5.67	7.41	8.54
T_(UVA)_ (%)	6.06 ± 0.68	3.34 ± 0.36	2.44 ± 0.8	2.24 ± 0.07	0.76 ± 0.15	0.53 ± 0.17
T_(UVB)_ (%)	5.29 ± 0.69	2.75 ± 0.36	1.8 ± 0.82	2.25 ± 0.07	0.9 ± 0.10	0.61 ± 0.23
UPF	23 ± 2.75	29 ± 2.45	>40	>50	>50	>50
